# Effectiveness of Oral Azithromycin in Treating Enteric Fever: A Hospital-Based Study on Pediatric Patients

**DOI:** 10.7759/cureus.67024

**Published:** 2024-08-16

**Authors:** Afzal Khan, Inayatullah Khan, Ahmad N Babar, Yasir Khan, Gulmina Shah, Muhammad I Khan

**Affiliations:** 1 Pediatrics, Lady Reading Hospital Medical Teaching Institution (MTI), Peshawar, PAK; 2 Pediatric Medicine, Lady Reading Hospital Medical Teaching Institution (MTI), Peshawar, PAK; 3 Gastroenterology, Lady Reading Hospital Medical Teaching Institution (MTI), Peshawar, PAK

**Keywords:** xdr salmonella, infectious disease, children, enteric fever, azithromycin

## Abstract

Introduction

Enteric fever is prevalent in underdeveloped and developing countries. It is caused by Salmonella Typhi, which has developed resistance over the years to commonly used antimicrobials. Meropenem is an effective treatment for all complicated and uncomplicated extensively drug-resistant (XDR) bacteria, but it is administered intravenously, three times daily, by infusion, and it is quite expensive for the patient. Oral azithromycin is shown by some authors to be effective in extensively drug-resistant enteric fever.

Material and methods

This retrospective cross-sectional study was conducted in the outpatient department of Lady Reading Hospital Medical Teaching Institution, Peshawar. The duration of the study was one year. Data was collected after approval from the hospital's Ethical and Research Committee. All pediatric patients meeting the inclusion criteria for extensively drug-resistant enteric fever were included. Data on patient demographics, blood culture and laboratory results, treatment given, and effectiveness were documented in a specialized proforma. Statistical Package for Social Sciences (SPSS) version 23 (IBM Corp., Armonk, NY, US) was used for data analysis.

Results

Out of the total 106 patients, 72 (67.9%) were male and 34 (32.1%) were female. The mean age was 7.51 ± 2.75 years, with a range of 1 to 15 years. Among them, 66 (62.3%) had anemia (hemoglobin less than 11 grams per deciliter for under 5 years and 11.5 for 5-15 years old children), with a mean hemoglobin level of 10.6 ± 1.53 grams per deciliter (g/dl), ranging from 7.2 to 13.8 g/dl. Thrombocytopenia was found in 14 (13%) patients. The mean platelet count was 317 x 10^3^ ± 164 cells per microliter, with a range of 61 x 10^3^ to 834 x 10^3^ cells per liter. The mean total leukocyte count was 9.71x 10^3 ^± 4.321 cells per microliter, with a range of 2.01 x 10^3^ to 30.40 x10^3^ cells per microliter. However, leucopenia was seen in only 5 (4.7%) patients. In 98.1% of cases, azithromycin was found to be effective in treating enteric fever caused by extensively drug-resistant Salmonella.

Conclusion

Azithromycin is effective in treating extensively drug-resistant enteric fever. It can be confidently used in patients with no or mild complications with extensively drug-resistant enteric fever. Good compliance and complete dosage should be followed to avoid resistance to this drug. Blood cultures should always be sent when prescribing antibiotics, especially when suspecting enteric fever.

## Introduction

Typhoid fever is primarily caused by the Gram-negative pathogen Salmonella Typhi and poses a significant burden on global health systems. In 2017, there were 14.3 million cases of typhoid and paratyphoid fever, resulting in 135,900 deaths [1]. Generally, a substantial portion of these cases occurs in Asia, which is considered the hub of enteric fever. Studies have found that the adjusted incidence in South Asian countries exceeds the threshold for a “high burden” of enteric fever (100,000 person-years) [2].

The first effective treatment for typhoid fever was chloramphenicol, introduced in the late 1940s [3]. Other first-line medications, such as co-trimoxazole and ampicillin, were used in the 1970s. However, resistance to these medications soon emerged. Subsequently, in the 1990s, fluoroquinolones were introduced and were found to be highly effective in treating Salmonella infections [3].

With good hygiene, vaccination, and effective antimicrobial agents, particularly fluoroquinolones and third-generation cephalosporins, enteric fever was not initially considered a major threat to health systems [4]. Unfortunately, this changed in 2016 when an outbreak of fluoroquinolone- and cephalosporin-resistant Salmonella was identified in Hyderabad, Sindh, and Pakistan. Similar cases of extensively drug-resistant (XDR) Salmonella, resistant to many antimicrobial agents except carbapenem and azithromycin, were reported in India, Bangladesh, and Nepal [5].

Several studies have reported the prevalence of XDR Salmonella in different contexts. In Lahore, Pakistan, a study found that 34.4% of Salmonella Typhi isolates were classified as XDR, with the predominant resistance gene being ampC [6]. In Beijing, China, an outbreak of XDR Salmonella Typhi highlighted the ongoing threat of this strain [7]. Similarly, a study conducted in Karachi, Pakistan, focused on the clinical course and treatment response of XDR Salmonella Typhi in pediatric patients, finding that azithromycin alone or in combination with meropenem was effective in treating the infection [8].

The prevalence of XDR typhoid fever has increased, leading to limited treatment options [9]. XDR Salmonella Typhi isolates in Pakistan have acquired antibiotic resistance genes, including those for first-line drugs (blaTEM-1, catA1, sul1, and dhfR7) and second-line drugs (gyrB, gyrA, qnrS, ParC, and ParE) [9]. Additionally, the presence of CTX-M genes (ESBLs) has rendered these isolates resistant to third-generation cephalosporins [10]. The current empiric choice for treatment, i.e., azithromycin, is also vulnerable to resistance [11]. The emergence of azithromycin resistance in XDR Salmonella Typhi is distressing and requires careful monitoring in endemic countries like Pakistan. The burden of XDR typhoid in Pakistan is significant, with a high number of XDR Salmonella Typhi isolates identified in blood culture samples [11].

Azithromycin, a broad-spectrum antibiotic, has shown promising results as a potential treatment for XDR Salmonella infections [12]. It belongs to the Macrolide class of antibiotics and works by inhibiting bacterial protein synthesis. Studies have indicated that azithromycin can effectively target and kill XDR Salmonella strains, even those resistant to other antibiotics. Furthermore, its ability to penetrate cells and tissues makes it an attractive option for treating systemic infections caused by XDR Salmonella. However, further research and clinical trials are needed to determine the optimal dosing regimen and evaluate its long-term effectiveness [9,11,12].

In the context of XDR Salmonella infection and its high prevalence, it is challenging for developing countries to cope with costly medications. The purpose of the study is to determine the effectiveness of azithromycin in children with XDR Salmonella treated in pediatric outpatient settings. This will not only improve our decision-making and confidence in treating typhoid fever with an effective drug but will also contribute to hospital guidelines for antibiotic stewardship.

## Materials and methods

Operational definitions for this study are given in Tables 1-2.

**Table 1:** Operational definitions Sources: [3,13] XDR: extensively drug-resistant

Title	Definition
Enteric Fever	A child was considered to have an enteric fever if they had the following features: Fever of more than 38°C, duration of fever of more than 4 days, no clinical focus of fever, and blood culture yields Salmonella.
XDR Enteric Fever	A child was considered to have extensively drug-resistant enteric fever if they had all the features of enteric fever and blood cultures yielded Salmonella resistant to most antibiotics except azithromycin and/or carbapenem.
Effectiveness	Treatment was considered effective if the patient responded, became afebrile by day 5, and did not require admission or carbapenem for the completion of enteric fever.
Azithromycin	It was used at a dose of 20 mg/kg/day to a maximum of 500 mg/day, once a day for a period of 7 days.

**Table 2:** Operational definitions for hematological variables Source: [14,15]

Hematological Variables	Definition
Anemia	A child will be considered to have anemia if his/her age is 1 to 5 years and hemoglobin is less than 11 grams per deciliter or age is 5 to 15 years and hemoglobin is less than 11.5 grams per deciliter.
Thrombocytopenia	A child will be considered to have thrombocytopenia if his/her platelet count is less than 150,000 cells/microliter.
Leucopenia	A child will be considered to have leucopenia if his/her white blood cell count is less than 4000 per microliter.

Study design

This is a retrospective cross-sectional study.

Setting

The study was conducted in the Outpatient Pediatric department of Lady Reading Hospital Medical Teaching Institution, Peshawar.

Duration of study

The study included all patients diagnosed with culture-proven enteric fever and treated as outpatients from January 1, 2023, to December 29, 2023.

Table 3 presents the inclusion and exclusion criteria for patient selection.

**Table 3:** Inclusion and exclusion criteria for the selection of patients

Inclusion Criteria
i. All pediatric patients aged less than 15 years fulfilling the criteria of XDR enteric fever
ii. Patients were treated as outpatients with oral azithromycin
iii. Both genders
Exclusion Criteria
i. Patients who were already started on carbapenem
ii. Patients who were already started on azithromycin
iii. Patients with complications who needed admission and were treated as inpatients
iv. Patients with other diagnoses of febrile illness
v. Patients with mixed growth in blood culture
vi. Patients treated with intravenous azithromycin as inpatients

Data collection procedure

This hospital-based retrospective study was conducted after approval from the Institutional Review Board (IRB No: 173/LRH/MTI). Patient details from the Outpatient department's patient records and the Human Management Information System (HMIS) were searched. Data of all pediatric patients meeting the inclusion criteria for XDR enteric fever, who visited the Outpatient department with febrile illness, were assessed in the consultant clinic for suspected enteric fever, had laboratory workup including blood culture performed, and were treated with azithromycin as an outpatient. Data on these patients' demographics, blood culture, and laboratory results at initial presentation, treatment given, and effectiveness (response to treatment with azithromycin) were documented in a specialized proforma.

Data analysis procedure

The data were entered into the Statistical Package for Social Sciences (SPSS version 23; IBM Corp., Armonk, NY, US) for analysis. Mean ± standard deviation was calculated for numerical variables like age, hemoglobin level, leukocyte count, and platelet count. Frequency and percentages were calculated for categorical variables like gender. The effectiveness of azithromycin was calculated. All results were presented as tables/charts.

## Results

**Figure 1:**
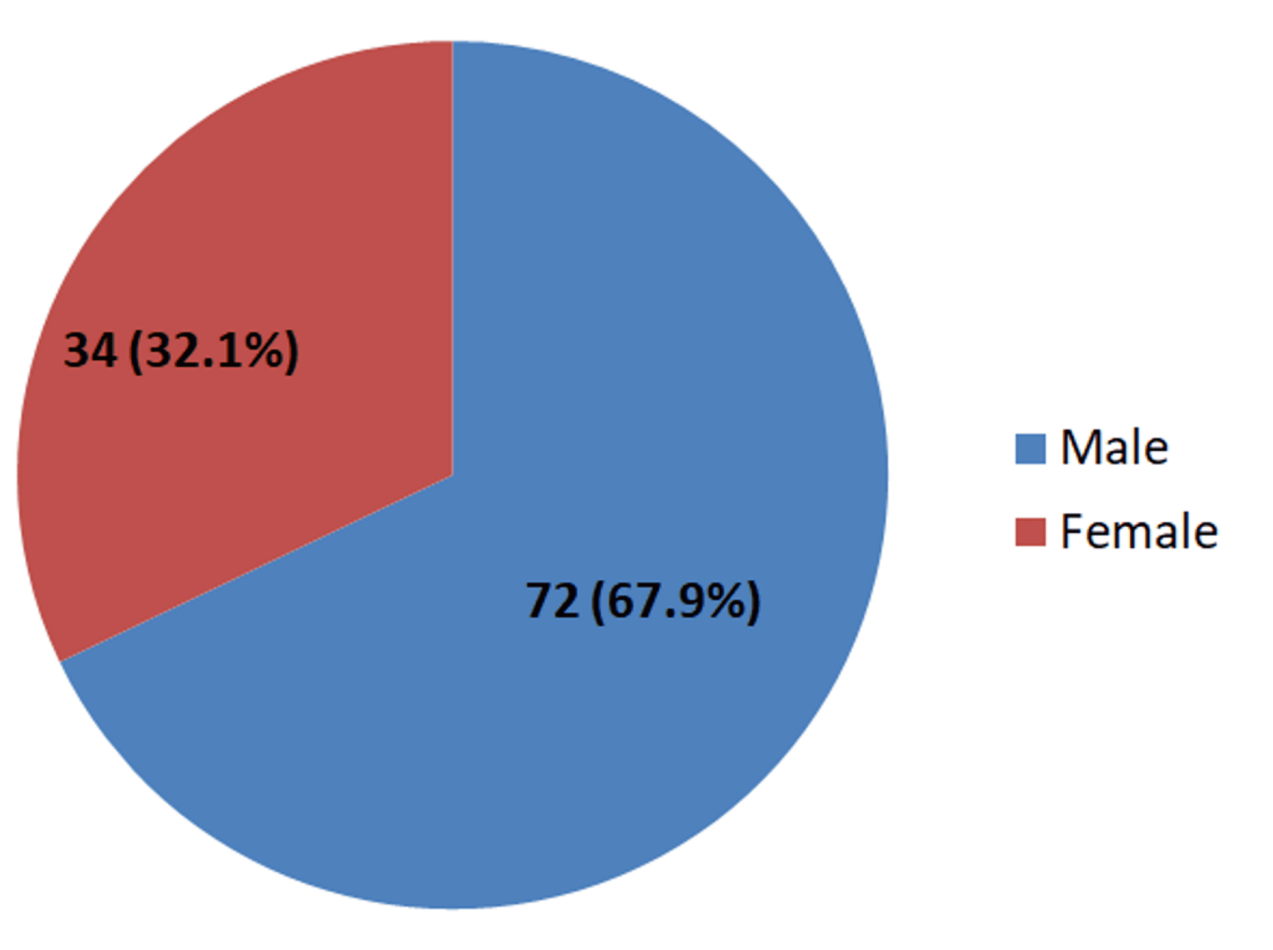
Gender-wise distribution of patients with enteric fever (n=106)

A total of 106 patients met our criteria and were included in our study. Of these, 72 (67.9%) were male and 34 (32.1%) were female (Figure 1).

The mean age was 7.51 ± 2.75 years, with a range of 1 to 15 years (Table 4).

**Table 4:** Age-wise distribution of children with enteric fever (n=106)

S. No	Age in years	Frequency	Percentage %
1	1-5	24	22.6
2	5.1-10	67	63.2
3	10.1-15	15	14.2
4	Total	106	100

Among them, 66 (62.3%) had anemia, with a mean hemoglobin level of 10.6 ± 1.53 grams per deciliter, ranging from 7.2 to 13.8. Thrombocytopenia was found in 14 (13%) patients. The mean platelet count was 317 x 103 ± 164 cells per microliter, with a range of 61 x 103 to 834 x 103 cells per microliter. The mean total leukocyte count was 9.71x103± 4.321 cells per microliter, with a range of 2.01x 103 to 30.40 x 103 cells per microliter. However, leucopenia was seen in only five (4.7%) patients (Table 5).

**Table 5:** Hematological profile of children with enteric fever (n=106)

S. No	Characteristics	Frequency	Percentage %	Mean±SD
1	Anemia	66	62.3	10.6 ± 1.53 g/dL
2	Thrombocytopenia	14	13	317x10^3^± 164/µl
3	Leukopenia	5	4.7	9.71x10^3^ ± 4.321/µl

**Figure 2:**
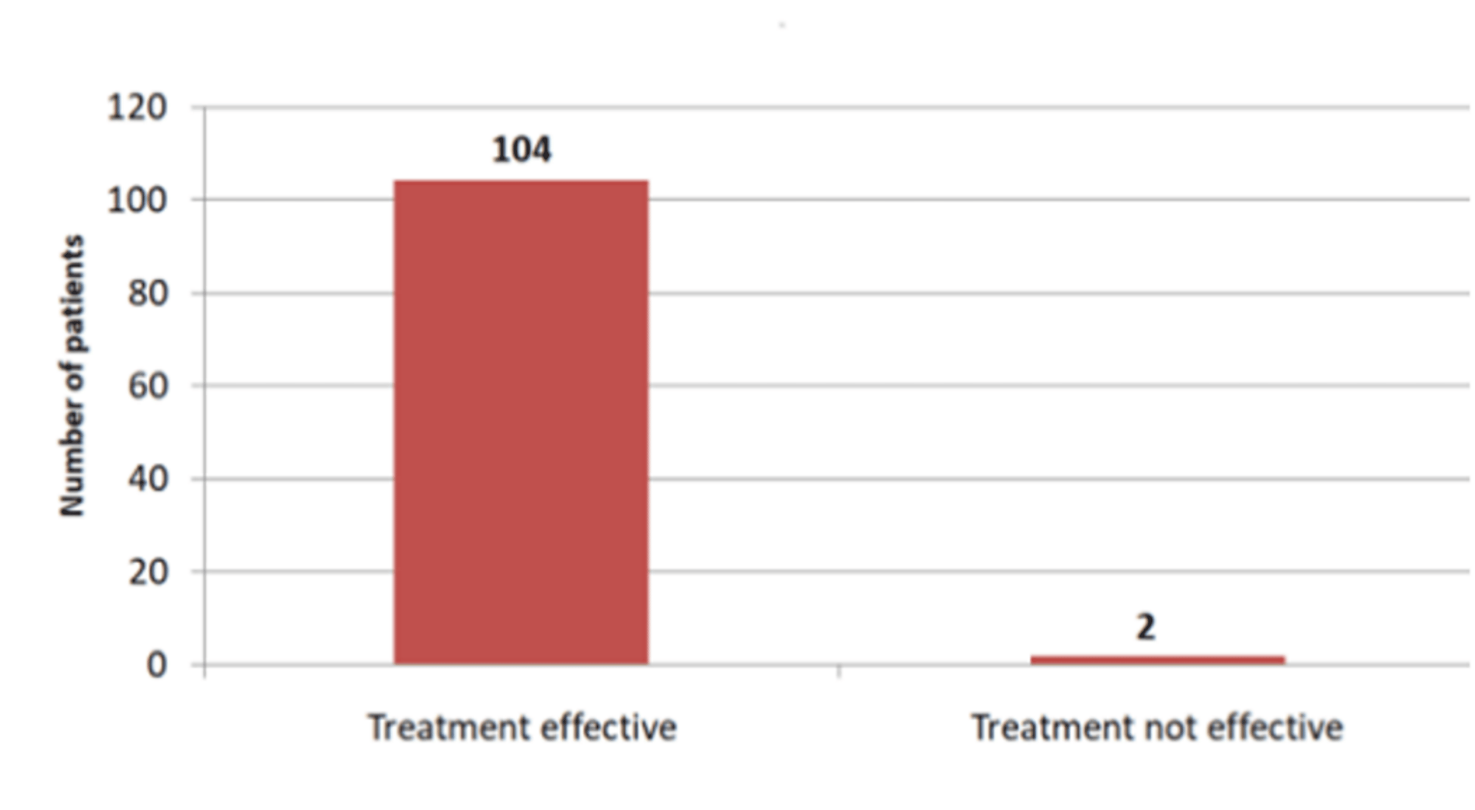
Effectiveness of azithromycin in children with XDR enteric fever (n = 106) XDR: extensively drug-resistant

In 98.1% of cases, azithromycin was found to be effective in treating enteric fever caused by extensively drug-resistant Salmonella (Figure 2).

## Discussion

The rise of XDR Salmonella has significantly increased the burden on health systems, particularly in resource-limited countries like Pakistan [16,17]. Resistance has evolved in this region to all first- and second-line antibiotics used to treat Salmonella infections. Many researchers have shown resistance of Salmonella to chloramphenicol, amoxicillin, trimethoprim-sulfamethoxazole, ciprofloxacin, and ceftriaxone [18-20]. Qamar FN et al. reported that in November 2016, there was an outbreak of ceftriaxone-resistant Salmonella Typhi in the Hyderabad region of Pakistan, with 486 individuals infected [5]. This resistance has since spread to many areas of the country [21,22].

Our study included a total of 106 patients diagnosed with blood culture and treated with azithromycin. Of these, 72 (67.9%) were male and 34 (32.1%) were female. The mean age was 7.51±2.75 years, ranging from 1 to 15 years. A total of 62.3% had anemia with mean hemoglobin being 10.6 ± 1.53 g/dl, ranging from 7.2 to 13.8. The mean platelets counts were 317x103± 164/µl with a range of 61x103 to 834x103. Thrombocytopenia was observed in 13% of the patients. The mean total leukocyte count was 9.71x103 ± 4.321/µl with a range of 2.01x103 to 30.40x103. Leucopenia was seen in only 4.7% of the patients. Azithromycin was found effective in treating enteric fever caused by XDR salmonella.

Our study results are comparable in demographics to those of other researchers. Ali et al., in their work on cephalosporin resistance in enteric fever, found consistent results with ours: 64% were male and 36% were female [23]. A similar gender distribution was shown by McCann N et al. in their study [24]. However, their work does not describe the hematological profile and resistance status of Salmonella to cephalosporin. Tahir N et al. reported that 51% of patients with enteric fever had anemia, 54.3% had thrombocytopenia, and 44.3% had leucopenia [25]. The high incidence of cytopenias in these patients is likely due to the study being conducted in a hematology department. Similarly, our study was performed on outpatients who were stable and less likely to have these complications. Patients with complicated enteric fever are admitted and are not part of this study. However, the mean age and gender distribution were comparable to our study, with a mean age of 8.17± 2.92 years and 48.6% male and 41.4% female [25].

Qureshi et al. found in their study that there was no significant difference in patients treated with oral azithromycin, intravenous meropenem alone, or a combination of intravenous meropenem and azithromycin [26]. However, the treatment cost was significantly lower at $5.87 for azithromycin compared to $88.46 for intravenous meropenem, suggesting an affordable and effective treatment option for resource-limited settings [26]. McCann et al. reported one treatment failure with azithromycin as an outpatient [24]. However, their study shows that Salmonella was sensitive to intravenous cephalosporin (ceftriaxone). Hussain et al. also found azithromycin to be effective against XDR Salmonella [27]. However, in their study, the number of patients treated with azithromycin was small, only 10 patients. In the study by Qureshi et al., one patient experienced treatment failure in the azithromycin group [26]. Similarly, Iqbal J et al. reported evolving azithromycin resistance in XDR Salmonella in Pakistan, which is a significant concern, as it will affect the management of enteric fever in outpatient departments [28].

The CDC (Centers for Disease Control and Prevention) recommends that enteric fever be considered in patients with acute febrile illnesses. Any patient who has visited Pakistan or Iraq in the last 30 days before the illness started may have acquired XDR Salmonella infection and be prescribed carbapenem if they have a severe or complicated disease, or azithromycin as empirical treatment [29]. WHO (World Health Organization) supported the XDR National Task Force in Pakistan in the development of an action plan on antimicrobial resistance. WHO advises testing microbiologically Salmonella Typhi and resistance patterns in patients suspected of enteric fever. It also considers azithromycin a reliable and affordable treatment option for resource-limited settings [30].

Limitations of our study include that it is a single-center, hospital-based design and there is a lack of community sample collection or sources of infection like water samples. Only clinically stable patients with no or mild complications were included. High-tech investigations for Salmonella Typhi serotypes and mutations were neither used nor available. The diagnosis was clinically suspected and then confirmed with a blood culture.

## Conclusions

Azithromycin is effective in treating XDR enteric fever with no or mild complications. Good compliance and complete dosage should be followed to avoid resistance to this drug. Blood cultures should always be sent when prescribing antibiotics, especially when suspecting enteric fever. We suggest that prospective clinical trials in the treatment of XDR enteric fever, especially in patients with complicated diseases, be designed to gain more evidence and confidence about the effectiveness of azithromycin. Additionally, vaccination and antibiotic stewardship should be implemented to combat this rising disease timely and effectively.
